# Predicting Food Safety Compliance for Informed Food Outlet Inspections: A Machine Learning Approach

**DOI:** 10.3390/ijerph182312635

**Published:** 2021-11-30

**Authors:** Rachel A. Oldroyd, Michelle A. Morris, Mark Birkin

**Affiliations:** 1 Leeds Institute for Data Analytics, University of Leeds, Leeds LS2 9JT, UK; m.morris@leeds.ac.uk (M.A.M.); m.h.birkin@leeds.ac.uk (M.B.); 2 School of Geography, University of Leeds, Leeds LS2 9JT, UK; 3 School of Medicine, University of Leeds, Leeds LS2 9JT, UK; 4 Alan Turing Institute, London NW1 2DB, UK

**Keywords:** food safety, food environments, food hygiene, machine learning

## Abstract

Consumer food environments have transformed dramatically in the last decade. Food outlet prevalence has increased, and people are eating food outside the home more than ever before. Despite these developments, national spending on food control has reduced. The National Audit Office report that only 14% of local authorities are up to date with food business inspections, exposing consumers to unknown levels of risk. Given the scarcity of local authority resources, this paper presents a data-driven approach to predict compliance for newly opened businesses and those awaiting repeat inspections. This work capitalizes on the theory that food outlet compliance is a function of its geographic context, namely the characteristics of the neighborhood within which it sits. We explore the utility of three machine learning approaches to predict non-compliant food outlets in England and Wales using openly accessible socio-demographic, business type, and urbanness features at the output area level. We find that the synthetic minority oversampling technique alongside a random forest algorithm with a 1:1 sampling strategy provides the best predictive power. Our final model retrieves and identifies 84% of total non-compliant outlets in a test set of 92,595 (sensitivity = 0.843, specificity = 0.745, precision = 0.274). The originality of this work lies in its unique and methodological approach which combines the use of machine learning with fine-grained neighborhood data to make robust predictions of compliance.

## 1. Introduction

Patterns of national food consumption have changed dramatically in recent years. In the most recent wave of the Food and You survey, it was reported that 98% of the UK population consume food from takeaways and restaurants, and at least 43% do so on a weekly basis [1]. Although drivers of consumer behaviors are complex and multi-faceted, this change can be partially attributed to a proliferation of food outlets; equating to 34% between 2010 and 2018 [2]. With consumers eating fewer home cooked meals than ever before, the governance of food-serving businesses is increasingly important, especially considering that an estimated 60% [3] of 2.4 million annual cases of foodborne disease [4] are thought to be contracted whilst eating away from home. Overseen by the Food Standards Agency (FSA), local and unitary authorities (referred to as LA’s for the remainder of this paper) are responsible for enforcing hygiene standards within food businesses in the UK. However, LA resources are becoming increasingly stretched.

LA spending on food control reduced from £125 to £101 million [5] between 2013 and 2019. In 2019, severe delays in routine food outlet inspections were reported, whereby only 14% of LA’s achieved their inspection targets [5]. Furthermore, over 20,000 UK food outlets were not inspected in the five years between 2013 and 2015. This lack of governance is problematic. Not only are food businesses not receiving the required support, but critical health violations (CHVs), inappropriate procedures, and structural problems can go unchecked, exposing consumers to unknown and potentially dangerous levels of risk. To address these issues, the FSA have proposed a program of regulatory change, known as Regulating Our Future (ROF) [6]. To date, the ROF program is yet to be realized, but one of the main proposals is to outsource a proportion of food business inspections and audits to private third-party assurance providers. This proposal has received heavy scrutiny from many industry experts, who argue it provides LAs with a mechanism to ‘mark their own homework’, by choosing assurance providers suited to their own needs and not necessarily those of the consumer [7].

Given the scrutiny of the ROF program, there is a clear justification to explore alternative avenues which may alleviate pressures placed on LAs. The work in this paper provides a data-driven approach to identify high risk food outlets to prioritize inspections for newly opened businesses and those awaiting inspections. This work capitalizes on the theory that food outlet compliance is impacted by its geographic context, namely the characteristics of the area within which it is located. This study assesses the utility of machine learning approaches to predict non-compliant food outlets (with food hygiene rating scheme scores ≤ 2). Socio-demographic, business type, and urbanness data at the output area (OA) level were used to train the models. These include age data (percentages of individuals in categories 0–4, 5–14, 15–19, 20–24, 25–44, 45–64, ≥65), ethnicity data (percentages of Asian, Black, Mixed, White, and ‘Other’ individuals), deprivation data (percentage of rented households, overcrowded households, households without car access, and individuals who are unemployed), region, rural urban classification, and output area classification. This approach is novel; therefore, we reviewed literature which has identified associations between neighborhood characteristics and food hygiene compliance. We also considered studies employing machine learning approaches in the context of food safety, as both are relevant to this work.

### 1.1. Neighbourhood Demography and Food Safety

Many studies have examined associations between food safety and neighborhood characteristics [8,9,10,11]. These studies primarily aim to investigate patterns of critical health violations (CHVs), and socio-demographic characteristics such as deprivation and ethnicity. For example, in their work using geographic information systems (GIS) to track CHVs in retail facilities in Philadelphia, Darcey and Quinlan [10] found that food outlet prevalence was higher in deprived areas; however, the frequency of CHVs was lower. This study also found that establishments in predominantly Hispanic areas had an increased number of CHVs, suggesting that Hispanic populations are at higher risk of foodborne illness than other ethnicities. The authors also postulate that the findings could result from inspection bias, where underlying factors influence both the frequency of inspections and identification of CHVs.

Pothukuchi, Mohamed [11] also discuss the associations between socio-demographic neighborhood characteristics and CHVs. In Detroit, Michigan, this study found that food outlets in deprived areas and areas with primarily African American populations had an increased number of CHVs compared to other areas. Whilst the authors also hypothesize that these results may be a function of inspection bias, they discuss the possible roles of language problems, inexperience with food safety practices, and cultural differences. With regards to the latter, it has been suggested that some unsafe food handing behaviors are more prominent among certain cultural or ethnic groups. In their ‘Kitchen Life Study’ Wills et al. [12] found that Pakistani participants were more likely to wash raw chicken, although this behavior opposes official guidance, as they believe it sanitary to do so. Other examples of unsafe food handling behaviors which may be adopted by specific populations, include eating food after its ‘use by’ date or incorrectly storing or reheating food. These behaviors contradict official guidance and increase the risk of infection by a foodborne pathogen.

Studies undertaken by Darcey and Quinlan [10] and Pothukuchi, Mohamed and Gebben [11] indicate that food outlet compliance, specifically CHVs, are related to or impacted by their geographic context. However, both studies clearly state that the reasons for these associations are unclear and further fine-grained research is required to understand the complex interactions between compliance, neighborhood characteristics, and the micro–macro context of regulatory processes. Further to this, in a review of the literature relating to food safety risks for populations of low socioeconomic status and minority ethnic groups, Quinlan [13] echoes this sentiment, stating that more research is required to understand the socio-economic associations between foodborne illness incidence, retail access, and food handling behaviors.

Considering studies which have been undertaken in the UK, these often utilize the food hygiene rating scheme (FHRS), whereby every food serving outlet is awarded a score from zero to five following inspection by a public health official (aside from those located in Scotland where a pass–fail system operates). Scores of 3–5 represent ‘broadly compliant’ businesses and 0–2 represent ‘broadly non-compliant’ business (we use the terms ‘compliant’ and ‘non-compliant’ throughout this paper). In their work, Fleetwood et al. [3] used the FHRS data to find significant associations between low scoring food establishments and contaminated microbiological food samples. This suggests that FHRS scores can be used as a proxy for foodborne illness risk.

Oldroyd, Morris and Birkin [14] also used the FHRS to examine associations with neighborhood characteristics and compliance. Logistic regression was employed to identify socio-demographic, urbanness and business type determinants of non-compliant food outlets in England and Wales. Specifically, this work reported that food outlets located in the most deprived quintile were 25% less likely to meet hygiene standards compared to those in the least deprived quintile. Takeaways and small convenience retailers, alongside outlets in large conurbation areas, were also less likely to score a FHRS score of three or above compared to restaurants and food outlets in rural areas. Small but significant associations were also reported between non-compliance and increased prevalence of certain age-group categories and Black, Asian, Mixed and ‘Other’ ethnicities. The findings of this study, particularly that food outlets in more deprived areas and those with higher proportions of ‘non-white’ ethnicities, support those found by previous research. A review of the literature to date indicates that compliance, or lack of, is most likely influenced by spatial location and geographic context.

### 1.2. Machine Learning Approaches for Food Safety 

We now turn our attention to studies which have utilized machine learning methods to examine aspects of food safety. This will inform the methodological approach of this work. Considered a subset of artificial intelligence, machine learning is concerned with the ability of a system to undertake a specific task without being explicitly programmed to do so. Rather, patterns and inference are used to automatically learn algorithms and improve upon them without a given set of instructions [15]. In a scoping review of the literature relating to methods for monitoring foodborne illness, Oldroyd, Morris and Birkin [16] report that the majority of food safety studies using machine learning aim to rapidly detect outbreaks of foodborne disease or calculate incidence over a specific time interval. They do so by classifying consumer-generated data (CGD), such as social media data or restaurant reviews, to identify first-person reports of illness and filter spurious data records which do not contain symptomatic reports. These approaches are often adopted to address problems with national reporting data which is untimely and underestimates the true incidence of foodborne illness as underreporting occurs at both the General Practitioner (GP) and patient level [17]. CGD can be analyzed in near real time and can be used to quantify foodborne illness incidence more accurately. Most food safety machine learning studies utilize content analysis and none, to our knowledge, have explored compliance prediction using neighborhood level data; however it is important to consider existing methodological approaches and how they can inform this research.

Sadilek et al. [18] used support vector machine (SVM) to classify 3.8 million tweets gathered from restaurant visitors in New York over a four-month period. Using check-ins and geolocation data, this study identified restaurant patrons and monitored their subsequent tweets for symptomatic language, determining 480 potential cases of food poisoning which had not been reported via the Department of Health. This study states that one of the main problems associated with machine learning classifiers is class imbalance, whereby a supervised machine learning algorithm will favor assignment of unlabeled records to the majority class if the classes are extremely imbalanced. By assigning all records to the majority class, the simple accuracy metric is improved [19]. Sadilek et al. [18] therefore used a method of human-guided machine learning, whereby tweets belonging to the minority class, those reporting symptoms of foodborne illness, were actively provided to the model during the training phase. As the class of interest is often the minority, class imbalance is an important consideration for many applications, and so too is the use of a model metric which is suitable for imbalanced problems. This holds true for classification of FHRS scores where non-compliant outlets comprise the minority class.

Alongside the use of Twitter data, other studies have analyzed the utility of restaurant review data to detect first person reports of foodborne illness [20,21]. Harrison et al. [22] used a probabilistic classifier to analyze 294,000 data records collected from the restaurant review platform Yelp. Reviews containing the words ‘sick’, ‘vomit’, ‘diarrhea’, or ‘food poisoning’, where two or more persons were reported ill, and those with an incubation period ≥ 10 h were used for subsequent analysis. This study identified 16 cases of foodborne illness which had not been reported via official channels. The results were verified via phone interviews conducted by epidemiologists. Although this study highlights the utility of CGD to detect foodborne illness, the authors state that this approach requires significant time and resources. They also emphasize that the methods should be used to supplement traditional approaches, as opposed to replacing them.

Although many studies have reported promising results from the use of machine learning approaches to identify outbreaks of foodborne illness using occurrences of specific key words in consumer-generated data, none to our knowledge have explored the utility of predicting food outlet compliance in a UK setting using small area data. In this work we used openly accessible socio-demographic, business type, and urbanness data to identify non-compliant food outlets. We analyzed various sampling approaches to address class imbalance as reported by previous studies. We aimed to answer the following research questions: Can non-compliant food outlets be identified through machine learning approaches, and if so, which are the most effective algorithms and sampling strategies?

## 2. Methodology

Three supervised machine learning approaches were trained and tested with a view to predict non-compliant food outlets (FHRS ≤ 2) in England and Wales: linear SVM; radial SVM; and random forest. Although linear SVM and radial SVM are permutations of the same algorithm with a different kernel, we refer to them as different approaches for the remainder of the paper to ease interpretation.

**Figure 1:**
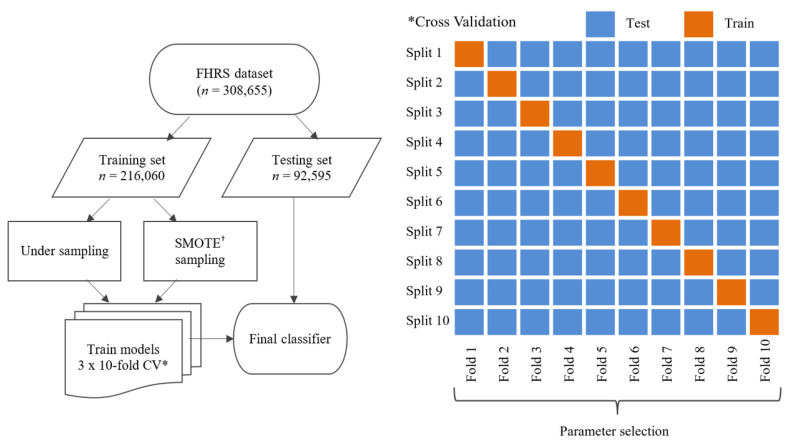
An overview of the analysis process. The full FHRS dataset is split into training and testing phases prior to SMOTE and under-sampling. ^†^ Synthetic minority oversampling technique. * Cross-validation.

An overview of the methodology can be viewed in Figure 1 and is as follows: prepare data, split dataset into training and testing sets, resample training set at different ratios using different strategies, train models using repeated cross-validation, apply algorithm to unseen testing set, calculate model metrics. We consider each step in further detail in the following sections.

### 2.1. Data and Data Preparation

Outcome and predictor variables can be viewed in Table 1. For the outcome variable, FHRS scores for individual food outlets in England and Wales were converted to a binary format where scores ≤ 2 were coded as 1 (‘non-compliant’, or the positive class) and scores ≥ 3 were coded as 0 (‘compliant’, or the negative class). These thresholds align with the FSA’s definitions of ‘not broadly compliant’ and ‘broadly compliant’ outlets, respectively. This format allows a binary classification to be undertaken. The FHRS scores represent hygiene standards at the time of the last inspection. The counts of scores across rating value categories are presented in the Supplementary Materials.

Predictor input features included: business type; region; rural and urban classification (RUC); output area classification (OAC); percentage of individuals in each age category; percentage of individuals in each ethnicity category; percentage of unemployed individuals; percentage of households with no car access; percentage of overcrowded households; and percentage of rented households. See Table 1 for full descriptions of variables and data sources. All input variables were collected at OA level, aside from business type and region which formed part of the FHRS dataset and were therefore reported for individual outlets. OAs were chosen as they represent the smallest statistical geography and are designed to be internally homogenous.

Business types which do not serve food to the immediate public were removed prior to analysis, including: hospitals, childcare centers, care homes; distributors, transporters; importers, exporters; farmers, growers; manufacturers, packers; schools, colleges, universities; mobile caterers. To ensure a sufficiently large number of data points in each category, the ten RUC categories were collapsed into five variables: urban cities and towns; rural hamlets and isolated dwellings; rural town and fringe; rural village; and urban conurbation. Categorical variables (business type; RUC; OAC; and region) were converted to binary dummy variables, where a column was created for each unique category. A value of 1 was assigned to indicate presence of the variable and 0 to indicate absence.

To attach neighborhood characteristics, each food outlet was matched to an OA code via its postcode using the Office for National Statistics Postcode to OA lookup [27]. In total, 99.7% of food outlets were matched to an OA code using this method and 0.3% of food outlets were discarded. The OA code was then used to join the aggregate OA level data to each food outlet. The final dataset comprised individual food outlets including their business type, FHRS and region, with OA level predictor variables attached, to represent the characteristics of the neighborhood. This allowed predictions to be made for individual outlets. 

All analysis was undertaken using the R statistical programming language, Vienna, Austria, v 5.3.1 [28]. Descriptive statistics for numerical predictor variables can be found in Table 2.

### 2.2. Dataset Partition 

The dataset (*n* = 308,655) was divided into training and testing sets with a 70:30 split, respectively. Stratified sampling was used to maintain the ratio of compliant and non-compliant establishments in each set. The largest proportion of the dataset was used to train the algorithms; the remaining 30% was set aside for testing performance. Whilst the training set was subject to different sampling strategies, the testing set was not resampled to ensure performance metrics were a true reflection of the model’s ability to predict the imbalanced dataset.

### 2.3. Sampling Strategy 

Given the imbalanced nature of the dataset, whereby the class of interest (non-compliant food outlets) comprises only 7% of the total dataset, the training dataset was resampled at several ratios using two different techniques. This would allow the optimal model to be determined.

#### 2.3.1. Undersampling

Five undersampled training datasets were created. These comprised of five ratios (1:1, 3:2, 2:1, 2:3, 1:2) of non-compliant to compliant outlets. These are referred to as sets 1–5 respectively. To create the undersampled training datasets, firstly, all non-compliant establishments in England and Wales (with a FHRS score ≤ 2) were selected (*n* = 14,226) from the parent training dataset. A randomly sampled subset of compliant outlets was then added to the non-compliant outlets. The number of which varied for each set based upon the required ratio. Under-sampling is the least resource-intensive sampling strategy as no additional computation is required; however, it can arguably lead to a weaker classifier due to the reduction of available training data.

#### 2.3.2. Synthetic Minority Oversampling Technique

Chawla et al. [29] suggest that the synthetic minority oversampling technique (SMOTE), whereby the minority class is oversampled whilst the majority class is undersampled, can achieve better classifier performance compared to under-sampling the majority class alone. To create synthetic points, the SMOTE method utilizes K-nearest neighbor to generate new data points, maximizing the amount of data available during the algorithm training process. For further information see Altman [30]. The DMwR package in R [31] was used to generate five SMOTE training datasets with the same class ratios as reported in Section 2.3.1.

### 2.4. Training Phase

Using both undersampled and SMOTE training datasets, a total of 33 models (five undersampled, five SMOTE datasets, and one unsampled dataset across the three algorithms) were trained using repeated 10-fold cross-validation (CV). The CV process is as follows: Each dataset is split into 10 equal folds using stratified sampling. A subset of the parameters across the 9 folds of data is used to fit the model and the tenth fold is used to compute performance metrics for that parameter subset. For each fold, the process is repeated three times. The area under the receiver operating characteristic curve (AUC) was computed for each cross-validation iteration and used to select the optimum input and tuning parameters. At the end of the training process the optimum model algorithm was reported.

To explain the AUC metric, we first considered the ROC curve which is a graph generated by plotting the proportion of correctly classified actual positives (in this case non-compliant outlets), known as the sensitivity, against the proportion of correctly classified actual negatives (compliant outlets), known as specificity, at various probability thresholds. There is often a trade-off between sensitivity and specificity whereby as one increases the other decreases. Therefore, a perfect ROC curve assumes the shape of a right angle, which passes through point (1,1) on the graph, indicating 100% specificity and sensitivity and maximizing the AUC. 

An AUC value of 1 indicates that 100% of the model predictions are correctly classified and a value of 0.5 indicates that only 50% of the classifications are correct, effectively allocating points at random. AUC accounts for both correctly and incorrectly classified data points, and is therefore considered superior to the accuracy metric when evaluating classifiers concerned with imbalanced classes [32].

### 2.5. Model Specifications

A brief overview of the three model algorithms is provided in the following sections.

#### 2.5.1. Linear Support Vector Machine

SVM is a non-probabilistic binary classifier which aims to find the optimum hyperplane between two classes in a 2D space. New data points are assigned to one of two classes depending on which side of the hyperplane they fall [33]. For further information see Vapnik [34]. In the Caret package in R, it is possible to impose a penalty for the misclassification of points during the training process, through the cost parameter. The higher the cost parameter, the lower the probability of the model misclassifying a point. We varied the value of the cost parameter throughout the training process using the tuneGrid function in Caret. The optimal and final value of which is reported in Table 3.

#### 2.5.2. Radial Support Vector Machine

In addition to performing linear classification, SVM can also perform non-linear classification by applying a Kernel Trick [35]. Whereby the model predictors are replaced with kernel functions. This enables the algorithm to operate in a high dimensional implicit feature space; for example, a 3D space. Coordinates of the data points in the newly transformed space are not explicitly calculated, which means this approach is more computationally efficient than others. Instead, the relationship between pairs of data is calculated [35]. Caret automatically tunes the cost parameter for radial SVM, the final values of which are reported in Table 3.

#### 2.5.3. Random Forest 

The third classification model we employ is random forest; an ensemble learning algorithm that averages the outcomes of several decision trees [36]. Combining multiple decision trees can address problems of overfitting, where individual classifiers often learn highly irregular patterns based upon training data resulting in low bias and extremely high variance, limiting their application beyond the training set. For ensemble learners, the variance of the overall model is decreased without increasing the bias, usually resulting in better performance than individual learners. 

Random forest differs from other ensemble learning methods as only a specific number of randomly sampled input features are available for each learner. The number of input features used in any one-fold of the cross-validation process is represented by the mtry argument; in Table 3 we report the final and optimal values for mtry. Commonly, learners opt for more predictive input features during training which can result in both overfitting and correlated outcomes between individual learners [37]. The advantage of choosing only a set number of random predictive variables is that those which appear highly predictive in the training set, but which are not in the testing set, are not oversampled during the learning process. For further information see Breiman [38].

### 2.6. Testing Phase

Following cross-validation whereby the models are fitted, their performance was assessed through the classification of unseen data points, the testing phase. Class probabilities were calculated for each unseen food establishment using the learnt algorithms and class labels were assigned using a probability threshold. The optimal probability threshold is the point on the ROC curve which maximizes both the distance from the diagonal and therefore the AUC, defined using Youden’s J statistic [39]. This is extracted using the coords function from the *pROC* package [40]. Once unseen data points were labeled, model metrics were calculated to test the model’s ability to predict non-compliant food outlets. Alongside AUC, sensitivity, and specificity, Cohen’s Kappa statistic and confusion matrices were also generated. A confusion matrix includes the number of true positives (TP), non-compliant food outlets correctly classified; false positives (FP), compliant food outlets incorrectly classified; false negatives (FN), non-compliant food outlets incorrectly classified; and true negatives (FN) compliant outlets correctly classified.

Cohen’s kappa statistic, henceforth referred to as kappa, provides a measure of agreement between two classifiers. More specifically, in supervised machine learning problems, kappa indicates the reliability of the generated class labels compared to the true labels, defined as:

(1) $$k=\frac{p_{o}-p_{e}}{1-p_{e}}$$

where $p_{o}$ is the observed accuracy or the number of correctly classified instances, and $p_{e}$ is the expected accuracy; the accuracy that a random classifier could expect to achieve by simply considering class frequencies alone. 

As kappa accounts for the number of instances in each class through inclusion of the expected accuracy metric, it is far more suited to the evaluation of imbalanced classification problems compared to the simple accuracy metric. Landis and Koch [41] provide a framework for the interpretation of kappa whereby they suggest a value < 0 indicates no agreement in actual and generated class labels, 0–0.20 indicates a slight agreement, 0.21–0.40 fair agreement, 0.41–0.60 moderate, 0.61–0.80 substantial, and 0.81–1 as almost perfect agreement. It is important to note that kappa must be interpreted on a case-by-case basis and alongside a confusion matrix indicating the number of true and false classifications for each class. An acceptable value of kappa will therefore differ depending upon the classification problem.

For the five top performing algorithms, the impact of assigning a cost weighting to the probability threshold was assessed. This acts as a penalty for false negative outcomes. In the seminal paper ‘The Foundations of Cost-Sensitive Learning’, Elkan [42] states “*Given a specification of costs for correct and incorrect predictions, an example should be predicted to have the class that leads to the lowest expected cost*” (Elkan, 2001, p. 973). As the cost of misclassifying a non-compliant outlet is much higher than misclassifying a compliant outlet, a relative cost of 30 was applied to reduce false negative labels. For weighted (applied cost penalty) probability thresholds, class labels were reassigned, and model metrics were recalculated to assess performance. Weighted and unweighted model metrics were compared.

Finally, for the best performing model, predictor variable importance was calculated using the varImp function from the Caret package in R. Importance scores for variables are generated by calculating the mean decrease in accuracy across trees when the variable is excluded. For more information see Liaw and Wiener [43]. The variable importance scores are scaled between 1 and 100 to aid interpretation. We reran the best performing model with the 20 most predictive variables and found that this did not alter the model metrics. Therefore, we decide not to undertake further feature selection. 

## 3. Results

In this section, we first consider ROC curves and AUC values to give an overview of the predictive power of the approaches across sampling strategies. For the top five performing models we then present model metrics for labels generated using weighted (applied cost penalty) and unweighted probability thresholds. Model metrics for all models are presented in the Supplementary Materials. Finally, we present predictor variable importance scores for the top performing model and consider the direction of association. We do not report the results of the CV process here; however, average model metrics for kappa and AUC across CV iterations can be viewed in the Supplementary Materials.

### 3.1. ROC Curve Analysis

Of the three algorithms and sampling strategies, random forest models trained with SMOTE data reported the best predictive power, achieving AUC values ranging from 0.859 and 0.873. SMOTE radial SVM models reported the second highest AUC values (AUC = 0.740–0.761) closely followed by undersampled random forest models (AUC = 0.715–0.718). Linear SVM models trained with both SMOTE and undersampled datasets reported the lowest AUC values at 0.608–0.698 and 0.660–0.696, respectively.

**Figure 2:**
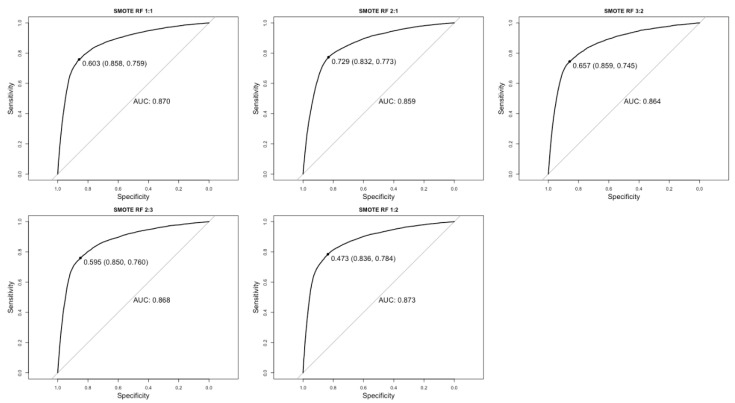
ROC curves for the five top performing algorithms. Random forest models trained using synthetic minority oversampling technique data at five sampling ratios. The diagonal line represents the AUC (Area Under Curve) of a random classifier.

A large range of AUC values are reported for SMOTE datasets across models (0.873–0.608); however, there was smaller difference in performance between the AUC values for undersampled datasets (0.718–0.660) indicating that sampling strategy has a greater influence over predictive performance than algorithm. ROC curves for the five top performing models are reported in Figure 2. See Supplementary Materials for all remaining ROC curves.

### 3.2. Application of Cost Penalty 

To further increase the sensitivity of the five top-performing random forest algorithms, and therefore classification of the non-compliant class, a cost penalty for false negative classifications was applied as described in Section 2.6. Weighted (applied cost penalty) and unweighted model metrics for the five SMOTE random forest models are presented in Table 4. Here we also include the results of the random forest model trained on an unsampled dataset. The size of the testing set remains constant to allow comparisons. 

As expected, application of the cost penalty lowers the probability threshold, resulting in an increase in the number of records classified as non-compliant. Although this strategy increases the number of FP classifications, it results in a large reduction of FN classifications, equal to 35%, 26%, 36%, 39%, and 30% for random forest sets 1 to 5, respectively. Sensitivity measures for weighted models are much higher (0.833–0.853) than unweighted sensitivity measures (0.745–0.784), reflecting an increase in the predictive power of the non-compliant class. 

Application of the cost penalty negatively effects the overall model metrics, with weighted models exhibiting lower AUC, kappa, and precision values compared to their weighted counterparts, where precision represents the fraction of non-compliant outlets among those labeled as such. However, in an applied setting, a reduction in FN classifications takes precedence over model metrics. Of all weighted classifiers, random forest set 4 reports the lowest number of FN classifications (895) and the highest sensitivity (0.853); however, this model also reports the highest number of FPs and the lowest kappa value (0.21), indicating a borderline slight/fair agreement between expected and observed values and a move towards a ‘catch-all’ approach compared to other classifiers. 

This ‘catch-all’ approach is clearly exemplified in the weighted model metrics for the random forest model trained with an unsampled dataset. Here the rate of non-compliant records remains equivalent to the original data at 7%. The probability threshold for this model is extremely low at 0.021; therefore, the model labels all data points above this probability threshold as non-compliant. Of 92,595 unseen records in the test set a total of 83,494 were labeled as non-compliant by the unsampled model and of these 77,591 were incorrect classifications with an overall precision of 0.071. Kappa was reported at 0.01, suggesting that this model performs only slightly better than a random classifier.

Of all classifiers, on balance random forest set 1 is adopted as the final classifier as it reports the highest kappa (0.230) and precision (0.192) values of the weighted models, indicating the lowest number of misclassifications of both classes whilst reporting low values of FN classifications (957).

### 3.3. Variable Importance Scores

**Figure 3:**
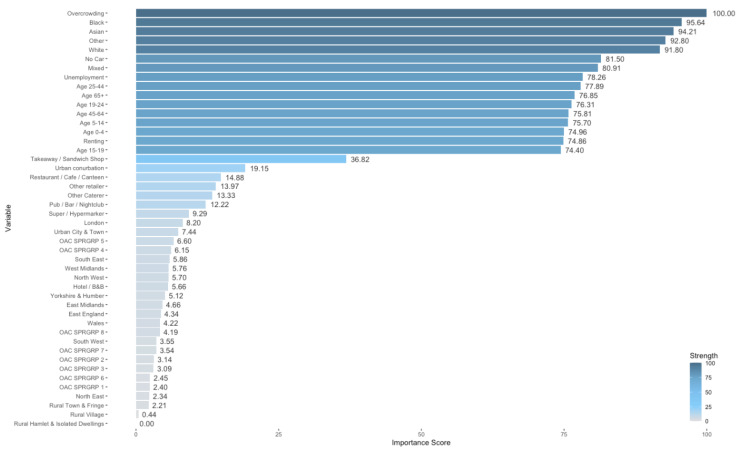
Variable importance scores for SMOTE random forest set 1 where red variables have higher predictive strength and blue variables have lower predictive strength.

**Figure 4:**
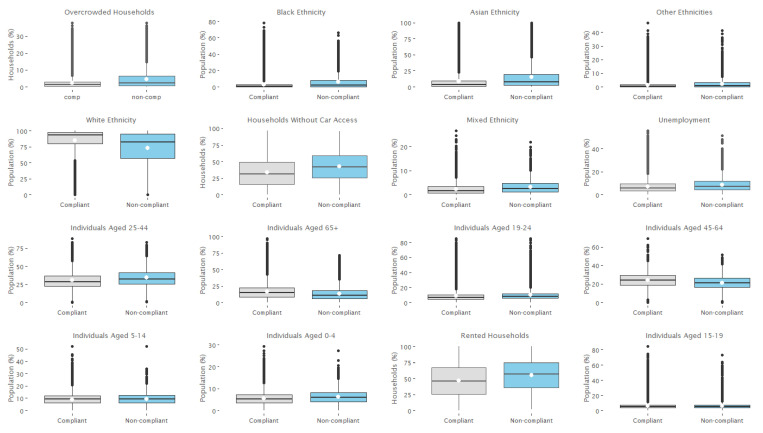
Boxplots for numeric predictor variables reported across the two classes.

Variable importance scores for SMOTE random forest set 1 predictor variables are presented in Figure 3. These are scaled between 1 and 100 to aid interpretation. Although we decide to calculate importance scores to further understand the prediction outcomes, there are limitations associated with entropy-based classifiers which we discuss further in Section 4.3. To understand the direction of association, boxplots were generated for the top 20 variables. These are presented in Figure 4. 

Overcrowding, with a score of 100, was reported as the most predictive variable for food establishment compliance in the SMOTE random forest set 1 model. This is closely followed by ethnicity categories, Black (95.64), Asian (94.21), Other (92.80), and White (91.80). Following the ethnicity categories, no car access scored 81.50, mixed ethnicity scored 80.9, and percentage of unemployed individuals scored 78.26. Age categories were also reported to be highly predictive with scores between 74.40 and 77.89. For other variables, we saw a large drop in the reported predictive power. Takeaways and sandwich shops showed mild predictive strength with variable importance score of 36.82, and all other business types, RUC, region, and OAC variables scored below 20. Rural hamlets and isolated dwellings were reported as the least predictive variable with a score of 0.

By examining boxplots for numeric predictor variables and the difference in median across the two classes (compliant and non-compliant food outlets), we can assess the direction of association. The majority of variables were negatively associated with non-compliance, i.e., the median for these variables was higher for non-compliant food outlets compared to compliant food outlets. These included: all non-White ethnicities, overcrowding, no car access, renting, unemployment, and age categories 25–44 and 19–44. The percentage of White individuals, those aged over 65, and between 45–64 were positively associated with non-compliance and exhibited a higher median for compliant outlets. The differences in class medians for age categories 0–4, 5–14, and 15–19 were <1 and therefore did not very greatly across classes. 

## 4. Discussion

Of the three models, we found that the random forest algorithm produced the strongest predictive classifier. Of the adopted sampling strategies, models utilizing SMOTE training data at a 1:1 ratio yielded the best results and outperformed models trained with undersampled data and SMOTE data at different ratios. Furthermore, we found that this sampling strategy greatly improved model metrics, and the frequency of FN predictions compared to unsampled data. In this section, we discussed the implications of our findings before discussing the model algorithms, sampling strategies, and variable importance.

### 4.1. Strengths and Policy Implications

This work presents a model which can make robust predictions of food outlet compliance using small-area socio-demographic, neighborhood, and business type data. To our knowledge, this is the first study to utilize such data for predictive purposes in the domain of food safety. This work is similar in some respects to the previously reviewed literature [8,9,10,11] which focused on determining associations between food outlet CHVs and both ethnicity and deprivation. However, we extended previous research by identifying high-risk food outlets, highlighting the intrinsic link between food safety and neighborhood characteristics.

Although the prediction of food outlet compliance does not help to meet the LA targets with regards to required inspections, it could reduce consumer risk by capturing high risk establishments earlier in the inspection cycle. Especially given that over 20,000 food outlets in the dataset have not been inspected within the last five years. It is clear how a classifier of this nature could advance current food inspection processes by prioritizing food outlets awaiting repeat inspections of those newly opened. However, as we discuss in Section 4.3, we must ensure that predictions of this nature do not enforce biases which may or may not already be present in the system. For a newly opened food outlet or one awaiting inspection, we envisage that predictor variables would be gathered at OA level using the postcode reported on the business registration documentation. Once complete with all required predictor variables, the data record would be used to classify the individual food outlet using the pre-learnt algorithm which would be assigned a risk rating depending on a pre-defined probability threshold. In an applied setting, we postulate that if our test set were to represent food outlets awaiting inspection, approximately 26,815 (28%) of 92,595 outlets could be prioritized for inspection by the LA. Of these inspections, approximately 5139 would result in a FHRS score of 2 or below, capturing 84% of the total number of non-compliant outlets. A total of 72% of outlets would be labeled as lower priority, easing the strain on local authority inspectors, and reducing consumer risk.

It should be noted that the figures reported by our model represent averages across LA’s in England and Wales and therefore the predictive strength of the model will vary geographically. In further work we will examine spatial variations in model predictions and predictor variable strength. The impact of varying probability thresholds, dependent upon different cost weighting, will also be assessed.

### 4.2. Appropriateness of Algorithms and Sampling Strategies

Overwhelmingly, we found that the classification problem cannot be solved by linear SVM. These models reported the lowest metrics across both SMOTE and undersampled datasets, with some linear models reporting equivalent performance to a random classifier. Radial SVM, which transforms data into a non-linear space, performed slightly better than its linear equivalent; however, the random forest algorithms reported the best predictive power for unseen data points. Therefore, future work will look at advanced learners such as gradient boosted decision trees which may further improve performance metrics.

Of sampling strategies, we found SMOTE to be the most effective for compliance prediction. Unlike undersampling, where a large proportion of the majority class is discarded resulting in the loss of important contextual information, SMOTE retains most of the data points in the majority class whilst adding synthetic points to the minority class. Subsequently, SMOTE training datasets are inherently larger than undersampled counterparts, which could account for differences in predictive strength between models. We found that compliance prediction using unsampled data was not possible. 

With regards to sampling ratios, models utilizing training sets which best reflect real-world ratios, i.e., those with a higher frequency of compliant food outlets, report higher model metrics than those with a higher frequency of non-compliant outlets. Models trained with a 2:1 non-compliant to compliant dataset report the lowest AUC values regardless of sampling strategy. Future work will look towards supplementing the minority class with additional data such as historical records of non-compliant food outlets to attain more data whilst maintaining representative ratios.

### 4.3. Variable Importance

Although we calculated variable importance to further understand the outcomes of our model, the way in which scores are calculated for entropy based classifiers mean they should be approached with caution [44,45]. Decision tree algorithms, such as random forest, attempt to reduce entropy at each division in the data. Variables with multiple levels or values inherently provide more flexibility for data partition compared to categorical variables and are subsequently afforded greater importance [46].

We clearly see this effect in the variable importance scores for our predictive model. Continuous numerical variables representing overcrowding, ethnicity, age, unemployment, no car access, and renting households have high predictive importance compared to categorical variables: RUC, region, OAC, and business type. These findings contrast those found previously by Oldroyd, Morris and Birkin [14] where large effect sizes were found for takeaways and sandwich shops, and major urban conurbations, alongside smaller but significant associations for Mixed, Asian, Black, and Other ethnicities. This study did however find a clear gradient of association between increased deprivation and non-compliance, where the Townsend deprivation score was used [47], comprising of overcrowding, unemployment, renting households, and households without car access. These variables proved highly predictive in our final model algorithm.

Further research is required to understand associations and potential relationships between highly predictive variables and non-compliance. Firstly, as we discuss in Section 1.1, such associations maybe a function of inspection bias. Darcey and Quinlan [10] discuss the influence of confirmation bias, which exists when inspection results align with a preconceived notion based on the type of neighborhood within which an outlet is located. Furthermore, Pham et al. [48] state that health inspectors report communication problems with business owners who do not speak English. Aside from inspection bias, another possible explanation for associations between neighborhood ethnicity and non-compliance is the role of ethnic cuisine. Some studies have reported higher numbers of CHVs at outlets serving ethnic cuisine [49,50,51,52]; however, it is unclear whether the location of such food outlets relates to the underlying population composition. For example, are Chinese restaurants more commonly located in areas with higher numbers of Chinese residents? Moreover, the relationship between unsafe food behaviors and ethnicity also warrants further research. As discussed in Section 1.1, Wills, Meah [12] found that some populations are more likely to undertake unsafe food behaviors in domestic settings but it is unclear whether these findings would translate into a commercial setting.

Considering associations between non-compliance and deprivation (overcrowding, no car access, unemployment, and renting households), in their work, Pothukuchi, Mohamed [11] hypothesized that associations between neighborhood poverty and CHVs may reflect inferior building infrastructure in poorer neighborhoods, resulting from lack of monetary resources. This work also considers the role of competition between economically weak businesses: “*The consistent effect of neighbourhood poverty on violations…may suggest either absence of competition or fierce price competition between economically weak businesses in ways that permit fewer resources to be devoted to food safety*” (Pothukuchi, Mohamed, and Gebben, 2008, p. 329). This hypothesis is an interesting one, especially considering competition in the UK context. Display of FHRS scores outside food premises is not mandatory in England and Wales (as it is in Scotland and Northern Ireland) and there is an argument that this therefore reduces the incentive for food businesses to improve their ratings. Without physical display of the FHRS, consumers cannot make an informed decision as to whether the food outlet poses a risk, or whether to eat at a higher rated nearby outlet. Furthermore, deprived populations often have little choice over where they can purchase food, stemming from low levels of relative car access (and high fuel prices) and low uptake of e-Commerce [53]. Therefore, sustained business from consumers, through lack of choice, may also sustain non-compliant practices.

Population transience, staff turnover, and business turnover is also an avenue that requires further investigation. Yapp and Fairman [54] found that business proprietors were less likely to send staff on food safety training courses if their business experienced high staff turnover. This could explain low lower likelihood of compliance in large urban areas and areas with more individuals who are more likely to migrate, such as certain age brackets. With regards to business turnover, newly established businesses are thought to be disadvantaged during the inspection process as they do not have comprehensive records of food safety compliance. Whilst undertaking street audits to validate FSA food establishment data, Wilkins et al. [55] found less agreement in urban areas compared to rural areas, which was attributed to a higher turnover of food businesses, which again supports the argument that outlets in large urban areas are less likely to comply with food hygiene standards.

As discussed, these importance scores should be interpreted with caution and in future work we will utilize more sophisticated post hoc model interpretation techniques such as Local Interpretable Model-agnostic Explanations (LIME) [56], with the aim to provide a higher degree of confidence in the variable importance scores of predictive variables, such that we can postulate more confidently about associations. We will also explore alternative approaches such as partial permutations [30] or growing unbiased trees [57]. These algorithms are designed to reduce bias towards continuous variables during variable selection. Future work will also undertake further fine-grained analysis of the composite measures that make up the FHRS scores, such as confidence in management scores and structural integrity scores, to further unpick the relationships between highly predictive variables and model outcomes.

### 4.4. Methodological Limitations

As with all studies utilizing area-based measures, there may be problems associated with the geographical scale of analysis. The Modifiable Areal Unit Problem arises when point data, such as individuals or households, are aggregated to polygons [58]. The size and shape of the polygon will determine the aggregated units and therefore different results maybe reported when using an alternative geography. We used a small geography to minimize the effect; however, this cannot be entirely mitigated without using individual level data.

Our analysis assumes that the FHRS data reflect current hygiene scores at food serving establishments across England and Wales. When calculating model metrics and the number of false and true positives and negatives. we used the FHRS data as a gold-standard measure. However, as only 14% of local authorities are up to date with their planned inspections, this measure may not entirely represent current food hygiene practices and our calculated metrics may under or overestimate model performance as a result. Furthermore, false positive predictions, i.e., food establishments predicted non-compliant but with a compliant FHRS score, may provide strong indication of where repeat inspections should be undertaken.

Our model is static and does not incorporate dynamic data which would contribute real-time information to improve the classification of non-compliant food outlets. We also did not include features relating to inspection history as these are not reported in the FHRS dataset; however, limited research has suggested that the inclusion of such data could help improve predictions. In future work we will incorporate CGD such as restaurant reviews and ratings, and we will also investigate the possibility of including individual outlet inspection data to further improve the predictive power of the model.

## 5. Conclusions

Using socio-demographic, business type, and neighborhood data, we determined a random forest model that can predict non-compliant food outlets in England and Wales with 84% sensitivity. We conclude that training the model using SMOTE data at 1:1 ratio is effective at addressing problems associated with highly imbalanced classes. We conclude that food outlet compliance is a function of geographic context by identifying highly predictive neighborhood features such as measures of deprivation, underlying population composition, and urbanness. To summarize, this data driven approach could be utilized to prioritize food outlet inspections for high-risk outlets, to deploy scarce LA resources more effectively and to reduce consumer exposure to unsafe food practices.

## Figures and Tables

**Table 1:** Data sources and variables.

Data Domain and Source	Geography	Variable	Categories/Levels
Food Hygiene Rating Scheme Scores [23]	Reported for individual food outlets	FHRS score (ordinal)	0 (Improvement necessary), 1, 2, 3, 4, 5 (Very good)
		Business Type (categorical)	Restaurants, cafés, & canteens; other retailers; super- & hyper-markets; other catering; pubs, bars, & nightclubs; takeaways & sandwich shops; hotels, guesthouses, bed & breakfasts
		Region (categorical)	East Midland, West Midlands, East of England, London, North East, North West, South East, South West, Wales, Yorkshire
Socio-demographic 2011 census data [24]	Reported at OA level	Age (% of persons)	0–4; 5–14; 15–19; 20–24; 25–44; 45–64; 65+
		Ethnicity (% of persons)	Asian, Black, Mixed, Other, White
		Unemployment (% of persons)	
		Overcrowding (% of households)	
		No car access (% of households)	
		Renting (% of households)	
Rural Urban Classification [25]	Reported at OA level	RUC (categorical):	Urban cities and towns; rural hamlets and isolated dwellings; rural town and fringe; rural village; and urban conurbation
Output Area Classification [26]	Reported at OA level	OAC Supergroups (categorical):	(1) Rural residents; (2) cosmopolitans; (3) ethnicity central; (4) multicultural metropolitans; (5) urbanites; (6) suburbanites; (7) constrained city dwellers; (8) hard-pressed living

(FHRS = Food Hygiene Rating Scheme Score, OA = Output Area, OAC = Output Area Classification, RUC = Rural Urban Classification).

**Table 2:** Descriptive statistics for numerical predictor variables. All variables are reported at output area level (SD = Standard Deviation).

Variable	Level	Mean	SD	Min.	Max.
Ethnicity (%)	White	84.06	19.64	0.00	100.00
	Mixed	2.42	2.31	0.00	26.61
	Asian	8.73	13.69	0.00	99.76
	Black	3.28	6.41	0.00	78.04
	Other	1.40	2.69	0.00	48.90
Age (%)	0–4	5.62	2.86	0.00	29.30
	5–14	9.10	4.49	0.00	52.20
	15–19	5.86	4.62	0.00	84.62
	20–24	9.13	8.35	0.00	85.12
	25–44	30.72	11.59	0.00	88.33
	45–64	23.74	7.96	0.00	69.19
	≥65	15.83	10.35	0.00	96.75
Unemployment (% of individuals)	-	7.35	5.53	0.00	55.68
Overcrowding (% of households)	-	2.57	3.76	0.00	38.00
No Car Access (% of households)	-	34.45	21.48	0.00	96.71
Renting (% of households)	-	47.53	24.77	0.00	100.00

**Table 3:** Final model tuning parameters. For linear and radial SVM, the cost parameter represents the optimal penalty threshold for misclassifications. For the random forest models, mtry, the optimal number of randomly selected predictor variables is reported (SVM = Support Vector Machine, SMOTE = Synthetic Minority Oversampling Technique).

Sampling Set/Ratio (Non-Comp:Comp)	Model Tuning Parameters					
	Linear SVM (Cost)		Radial SVM (Cost)		Random Forest (Mtry)	
	SMOTE	Under-Sampled	SMOTE	Under-Sampled	SMOTE	Under-Sampled
Set 1 (1:1)	1.895	0.842	32.00	32.00	5	3
Set 2 (2:1)	0.632	0.947	64.00	0.250	5	3
Set 3 (3:2)	0.316	0.105	64.00	2.000	6	3
Set 4 (2:3)	2.000	2.000	16.00	16.00	5	3
Set 5 (1:2)	1.368	2.000	16.00	0.250	5	3

(SVM = Support Vector Machine, SMOTE = Synthetic Minority Oversampling Technique).

**Table 4:** Weighted and unweighted performance metrics for random forest models utilizing SMOTE datasets across 5 sampling strategies where weighted observations have a cost penalty applied (30) when extracting the optimal probability threshold and where precision is the proportion of correctly classified non-compliant outlets.

Metric	RF Set 1 *n* = 92,595		RF Set 2 *n* = 92,595		RF Set 3 *n* = 92,595		RF Set 4 *n* = 92,595		RF Set 5 *n* = 92,595		RF Unsampled *n* = 92,595	
	Unweighted	Weighted	Unweighted	Weighted	Unweighted	Weighted	Unweighted	Weighted	Unweighted	Weighted	Unweighted	Weighted
Probability Threshold	0.603	0.481	0.729	0.645	0.657	0.515	0.595	0.459	0.473	0.367	0.067	0.021
Area Under Curve	0.87	0.87	0.859	0.859	0.864	0.864	0.868	0.868	0.873	0.873	0.796	0.796
Sensitivity	0.759	0.843	0.773	0.833	0.745	0.838	0.76	0.853	0.784	0.849	0.661	0.859
Specificity	0.858	0.745	0.836	0.741	0.859	0.737	0.85	0.724	0.836	0.737	0.797	0.481
True Positives	4624	5139	4712	5076	4540	5107	4630	5201	4781	5175	4029	5903
False Positives	12,264	21,676	14,572	22,383	12,180	22,752	12,976	23,872	14,210	22,773	17,571	77,591
True Negatives	74,235	64,823	71,924	64,116	74,319	63,747	73,523	62,627	72,289	63,726	68,928	8908
False Negatives	1472	957	1384	1020	1556	989	1466	895	1315	921	2067	193
Kappa	0.338	0.230	0.301	0.218	0.334	0.216	0.325	0.210	0.313	0.220	0.210	0.010
Precision	0.274	0.192	0.244	0.185	0.272	0.183	0.263	0.179	0.252	0.185	0.187	0.071

(SMOTE = Synthetic Minority Oversampling Technique, RF = Random Forest).

## Data Availability

Not applicable.

## Supplementary Materials

The following are available online at https://www.mdpi.com/article/10.3390/ijerph182312635/s1, Figure S1: Count of Food Hygiene Rating Scheme Scores in England and Wales. Figure S2: Box and whisker plots are generated for the Kappa metric averaged for cross-validation iterations. The minimum, maximum, mean and interquartile range are calculated for each model. Where US = under-sampled datasets; SMOTE = Synthetic Minority Oversampling Technique datasets; Linear = Linear SVM; Radial = Radial SVM; RF = Random Forest. Suffixed numbers represent sampling ratios of non-compliant to compliant food outlets as follows: 1 = 1:1; 2 = 2:1; 3 = 3:2; 4 = 2:3; 5 = 1:2. Figure S3: Box and whisker plots are generated for the AUC metric averaged for cross-validation iterations. The minimum, maximum, mean and interquartile range are calculated for each model. Where US = under-sampled datasets; SMOTE = Synthetic Minority Oversampling Technique datasets; Linear = Linear SVM; Radial = Radial SVM; RF = Random Forest. Suffixed numbers represent sampling ratios of non-compliant to compliant food outlets as follows: 1 = 1:1; 2 = 2:1; 3 = 3:2; 4 = 2:3; 5 = 1:2. Figure S4: ROC curves and AUC values are generated for each model. Steep ROC curves and high AUC values indicate better performance than shallow ROC curves and low AUC values. SMOTE Radial 1 and under-sampled Radial 3 did not converge. Table S1: Sensitivity, specificity, precision, and kappa for all models.
