# Supplementary material for: Predicting Food Safety Compliance for Informed Food Outlet Inspections: A Machine Learning Approach
Source: Int J Environ Res Public Health. 2021 Nov 30;18(23):12635. doi: 10.3390/ijerph182312635 (PMC8656817; doi:10.3390/ijerph182312635)
Supplement: Supplementary file 1 [file ijerph-18-12635-s001.zip › ijerph-1365176-supplementary.pdf]

International Journal of
Environmental Research and Public Health

Supplementary Material

Table S1. Sensitivity, specificity, precision, and kappa for all models.

Sampling Strategy		Model	Sensitivity	Specificity	Precision	Kappa
**SMOTE**	Linear SVM	1	0.670	0.604	0.109	0.020
		2	0.935	0.193	0.075	0.080
		4	0.720	0.555	0.102	0.070
		5	0.993	0.040	0.068	0.004
	Radial SVM	2	0.724	0.696	0.144	0.146
		3	0.735	0.691	0.143	0.146
		4	0.663	0.718	0.142	0.141
		5	0.728	0.655	0.130	0.122
**Under Sampling**	Linear SVM	1	0.652	0.641	0.114	0.092
		2	0.897	0.259	0.079	0.027
		3	0.898	0.262	0.079	0.027
		4	0.610	0.681	0.119	0.010
		5	0.598	0.684	0.118	0.098
	Radial SVM	2	0.648	0.644	0.114	0.092
		3	0.616	0.685	0.121	0.104
		4	0.603	0.703	0.125	0.111
	Random Forest	1	0.652	0.683	0.127	0.115
		2	0.623	0.701	0.128	0.116
		3	0.649	0.686	0.127	0.115
		4	0.658	0.673	0.124	0.111
		5	0.631	0.698	0.128	0.112

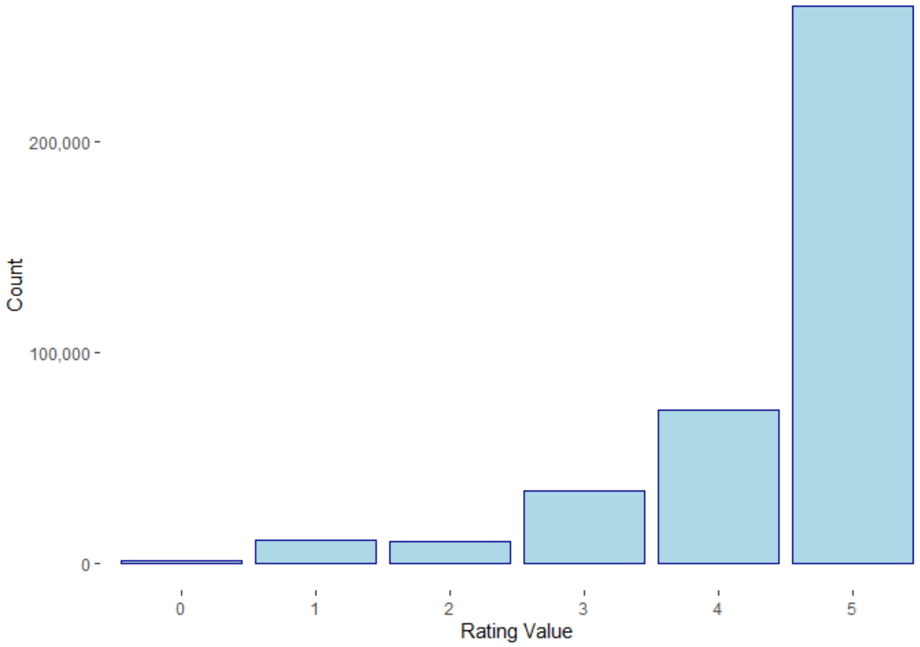

Figure S1. Count of Food Hygiene Rating Scheme Scores in England and Wales.

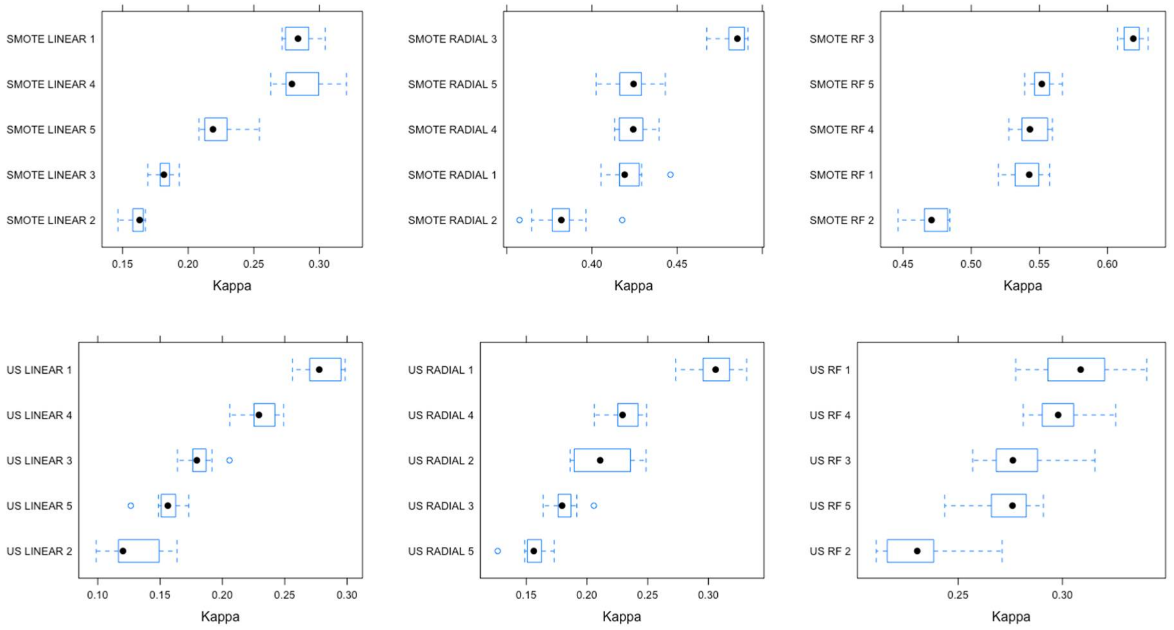

Figure S2. Box and whisker plots are generated for the Kappa metric averaged for cross-validation iterations. The minimum, maximum, mean and interquartile range are calculated for each model. Where US = under-sampled datasets; SMOTE = Synthetic Minority Oversampling Technique datasets; Linear = Linear SVM; Radial = Radial SVM; RF = Random Forest. Suffixed numbers represent sampling ratios of non-compliant to compliant food outlets as follows: 1= 1:1; 2= 2:1; 3= 3:2; 4= 2:3; 5= 1:2.

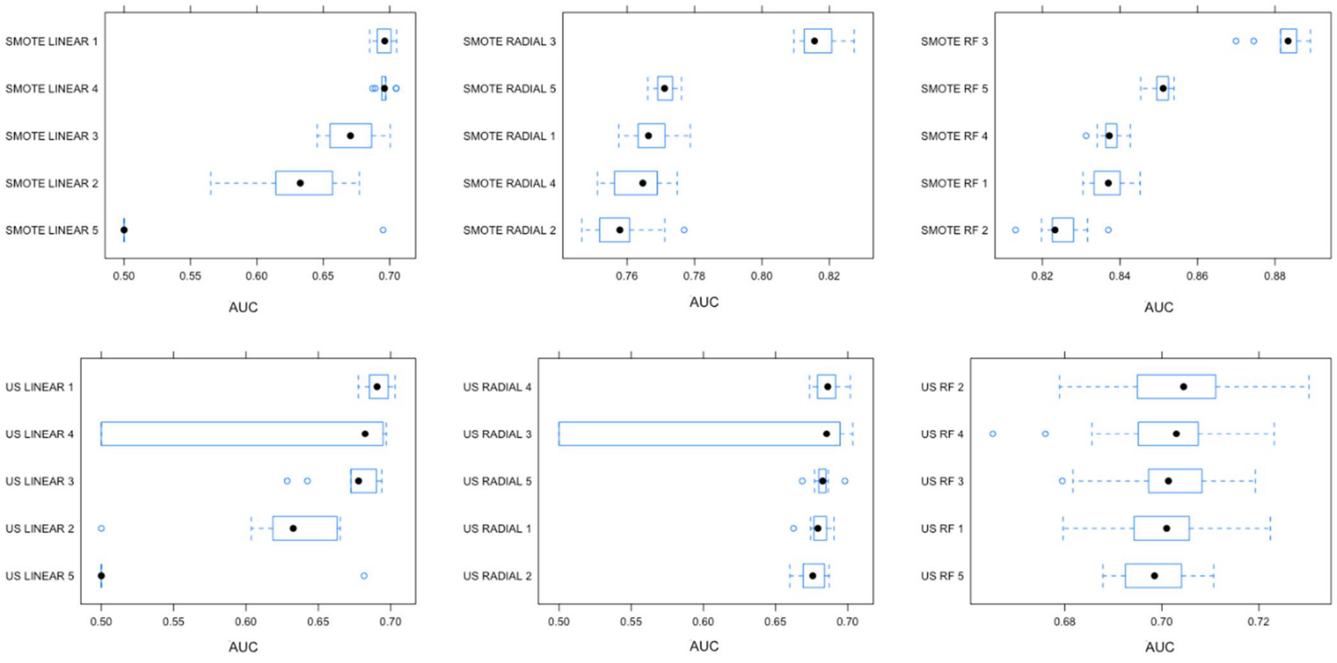

Figure S3. Box and whisker plots are generated for the AUC metric averaged for cross-validation iterations. The minimum, maximum, mean and interquartile range are calculated for each model. Where US = under-sampled datasets; SMOTE = Synthetic Minority Oversampling Technique datasets; Linear = Linear SVM; Radial = Radial SVM; RF = Random Forest. Suffixed numbers represent sampling ratios of non-compliant to compliant food outlets as follows: 1 = 1:1; 2 = 2:1; 3= 3:2; 4 = 2:3; 5= 1:2.

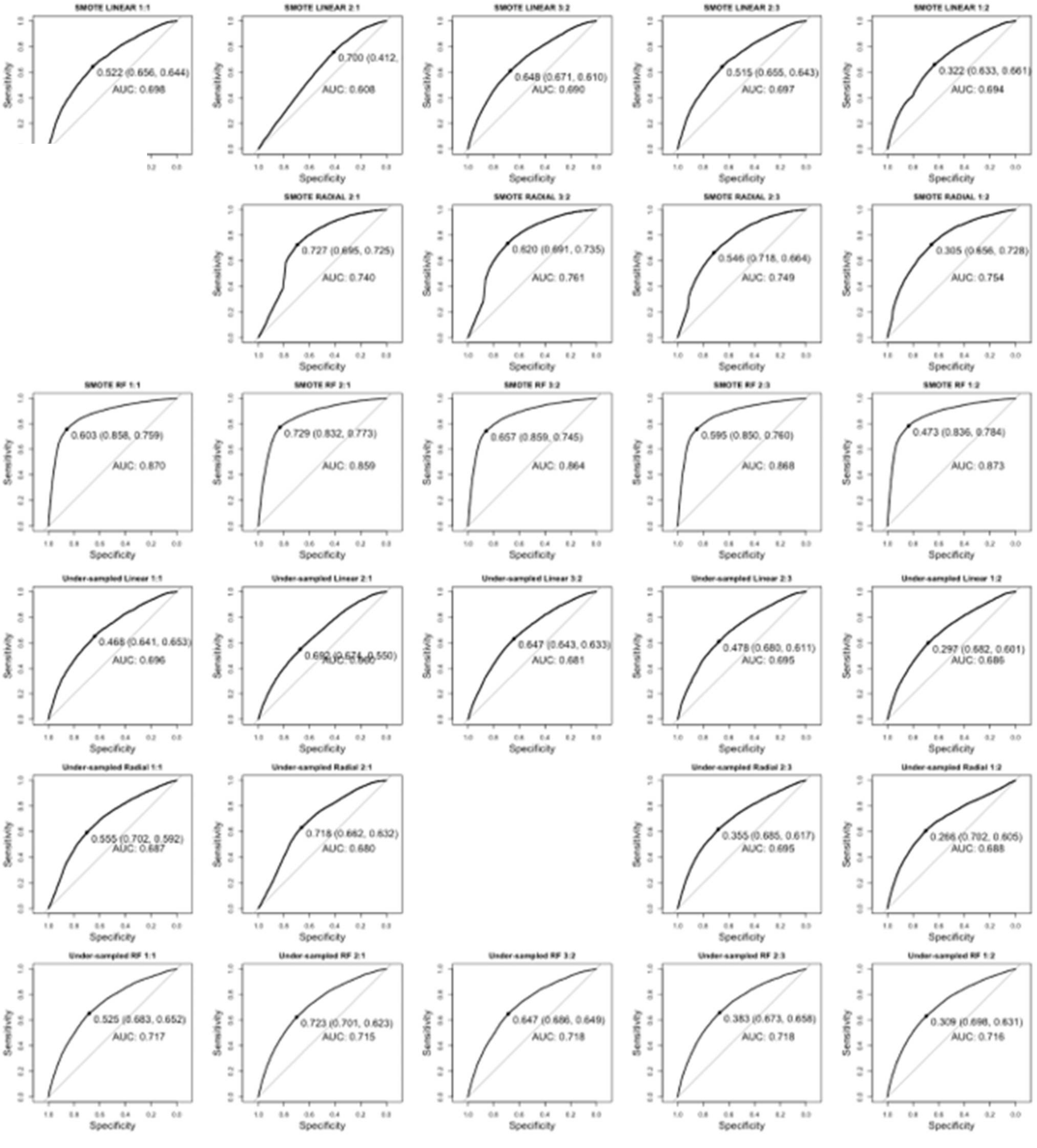

Figure S4. ROC curves and AUC values are generated for each model. Steep ROC curves and high AUC values indicate better performance than shallow ROC curves and low AUC values. SMOTE Radial 1 and under-sampled Radial 3 did not converge.
